# Evaluation of Relationship Between Neuromuscular Fatigue and Manual Dexterity in Physiotherapists: An Observational Study

**DOI:** 10.3390/brainsci16020193

**Published:** 2026-02-06

**Authors:** Gianluca Libiani, Francesco Sartorio, Ilaria Arcolin, Stefano Corna, Marco Godi, Marica Giardini

**Affiliations:** 1 Department of Physical Medicine and Rehabilitation, Istituti Clinici Scientifici Maugeri IRCCS, Institute of Veruno, Gattico-Veruno, 28013 Piedmont, Italy; gianluca.libiani@icsmaugeri.it (G.L.); stefano.corna@icsmaugeri.it (S.C.); marco.godi@icsmaugeri.it (M.G.); marica.giardini@icsmaugeri.it (M.G.); 2 Department of Medicine and Technological Innovation (DiMIT), University of Insubria, 21100 Varese, Italy; sartoriof60@gmail.com

**Keywords:** functional manual dexterity, physiotherapist, assessment, grip strength, workload, neuromuscular fatigue, perceived stress

## Abstract

**Highlights:**

**What are the main findings?**

• Functional manual dexterity, but not maximal grip strength, significantly declines by the end of the workweek in physiotherapists, and this can be reliably detected using the Functional Dexterity Test.
• Perceived stress, rather than sleep quality or workload, is associated with subjective fatigue, even early in the workweek, highlighting the central role of psychosocial factors in well-being.

**What are the implications of the main findings?**

• Fine motor performance may represent an early functional marker of neuromuscular fatigue in healthcare professionals, preceding overt losses in peak strength.
• Combining routine monitoring of manual dexterity and stress may provide a more comprehensive evaluation of physiotherapists’ functional readiness and occupational well-being.

**Abstract:**

**Background/Objectives:** Neuromuscular fatigue (NMF) can impair manual dexterity and strength in healthcare professionals. Due to their high physical and cognitive workloads, physiotherapists (PTs) are particularly susceptible to NMF. This study investigated whether NMF, expressed as changes in manual dexterity and grip strength, occurs over a workday and across a workweek in PTs, and explored its relationship with stress and sleep quality. **Methods**: A total of 43 full-time PTs (25 female, mean age 37.72 ± 11.94 years) were recruited. Manual dexterity was assessed using the Functional Dexterity Test (FDT), while maximal grip strength (MGS) was measured by a hand dynamometer. Reliability was evaluated on a subgroup using Intraclass Correlation Coefficients (ICC_3,1_) and Standard Error of Measurement (SEM). Evaluations were conducted at the beginning and at the end of the work shift, on Monday and Friday. Subjective fatigue, perceived stress, and sleep quality were also recorded. **Results**: The FDT showed excellent intra-rater reliability (ICC > 0.93; SEM < 0.94 s). FDT performance was significantly slower on Friday evening compared to all other time points (*p* < 0.01), exceeding the minimal detectable change thresholds. No significant changes were observed in MGS across the week. Perceived stress was strongly correlated with fatigue levels on Monday (ρ = 0.731) and Friday (ρ = 0.612) evenings. Sleep quality and professional experience did not correlate with performance changes. **Conclusions**: PTs experience a significant decline in manual dexterity by the end of the workweek, suggesting an accumulation of NMF. While MGS remains stable, fine motor control is more sensitive to fatigue. Psychosocial stress appears to be a major driver of perceived fatigue in this population.

## 1. Introduction

Neuromuscular fatigue (NMF), defined as the inability to sustain a motor task over time, is a common phenomenon that can substantially compromise daily functioning [1,2]. It affects athletes, older adults, and individuals with various medical conditions, and it is also highly relevant in occupational contexts [3,4]. In workers, NMF has a strong biopsychological component and should not be considered solely a mechanical failure of the neuromuscular system [5]. Rather, it is influenced by cognitive demands, stress, and psychological state, which play a central role in shaping both fatigability and the perception of effort [6].

In occupational settings, NMF can be broadly conceptualized as any acute impairment, such as reduced force production or increased perceived effort during task execution, regardless of whether task performance can still be completed successfully [7]. This condition negatively affects quality of life, work performance, and safety [8]. High workloads, limited recovery periods, strict deadlines, and repetitive, physically demanding tasks create environments that promote the onset and persistence of fatigue, which may progressively accumulate across the workweek.

Healthcare professionals frequently report working in a “fatigued” state, a condition likely associated with the high prevalence of work-related musculoskeletal disorders across multiple body regions [4]. Objective evidence of NMF has been documented among several healthcare professions. For example, post-shift emergency service nurses experience declines in manual dexterity and increases in perceived exertion, indicating that neuromuscular and mental fatigue during a work shift can impair fine motor skills in frontline healthcare professionals [9]. Surgeons performing complex procedures experience progressive mental fatigue over the course of the workday, with increases in reaction time and subtle impairments in attention and memory [10]. Furthermore, dental hygienists demonstrate reduced manual dexterity in both hands at the end of the working day, highlighting the vulnerability of occupations involving repetitive fine motor tasks [11]. Among healthcare workers, physiotherapists (PTs) may be particularly susceptible to NMF [12]. Their professional role extends beyond physical treatment delivery and includes sustained cognitive engagement, emotional labor, and continuous monitoring of patients with complex clinical needs. This combination of psychosocial demands and prolonged physical effort places substantial stress on their neuromuscular systems. Additionally, the frequent execution of manual techniques requiring precision and force, often performed for extended periods with limited rest, imposes a considerable load on the upper limbs, potentially impairing motor efficiency and endurance and ultimately affecting the quality of patient care [8].

Beyond job-related physical demands, perceived stress and sleep quality may substantially influence NMF and manual dexterity. Sustained exposure to stress increases cognitive load and attentional demands, leading to alterations in cortical processing and a reduced efficiency in central motor control during motor tasks [13]. Chronic psychological stress has been shown to decrease steadiness during isometric contractions, slow reaction times in visuomotor tasks, and increase perceived effort during repetitive fine motor activities, thereby exacerbating fatigability even during submaximal and precision-based tasks [5,6]. Importantly, increased central noise and reduced motor unit discharge coherence may preferentially compromise precision and coordination rather than maximal force output [1,5]. Similarly, sleep deprivation and poor sleep quality negatively affect motor performance. Marked sleep deprivation acutely impairs cortical and subcortical processing, reduces the efficiency of central motor drive, and disrupts motor unit discharge patterns, leading to immediate deficits in force steadiness, coordination, and precision-based tasks [14,15]. These effects tend to accumulate over time, becoming more pronounced with prolonged sleep disruption [16,17]. Taken together, these findings suggest that both chronic stress and sleep disruption could progressively degrade NMF and motor control across the workday or workweek.

Objectively assessing NMF remains challenging due to its multifactorial nature. Previous studies have quantified NMF through reductions in muscle strength, impairments in contractile function, or increases in perceived exertion [18]. However, fatigue may also manifest as alterations in electromyographic (EMG) activity or as deficits in fine motor control and manual dexterity [1,19]. These latter measures may be more sensitive than maximal strength testing for detecting subtle fatigue-related changes in neuromuscular control. Indeed, NMF has been shown to alter submaximal force production by increasing the amplitude and reducing the complexity of force fluctuations, changes that may not be captured by traditional strength measures alone. Nevertheless, maximal grip strength (MGS) remains one of the most widely used and clinically relevant indicators of overall neuromuscular function, as it reflects the integrity of central and peripheral neural drive, motor unit recruitment, and muscle contractile capacity [20,21]. Reductions in MGS have been consistently associated with neuromuscular impairments and functional limitations across clinical and occupational populations, supporting its use as a pragmatic surrogate measure of neuromuscular deficits [22]. Although EMG provides sensitive and objective insights into neuromuscular function, its use is often limited in routine clinical and occupational settings. Consequently, assessment tools that are simple, non-invasive, and do not require specialized instrumentation may offer greater translational value. In this regard, fine motor performance tests commonly used to assess manual and digital dexterity and precision, such as the Functional Dexterity Test (FDT) [23] or the Nine-Hole Peg Test [24], represent promising candidates for detecting fatigue-induced impairments in neuromuscular control in real-world contexts [25]. It should be noted, however, that in such applied settings, NMF is typically not measured through direct neurophysiological techniques, but is instead inferred from changes in functional motor performance that are compatible with fatigue-related mechanisms.

The primary aim of this observational study was to investigate whether functional signs of NMF, expressed as changes in manual dexterity and MGS, occur over the course of a workday and across a workweek in PTs. A secondary aim was to examine the relationships between objective performance measures and subjective factors, including perceived fatigue, stress, and sleep quality, as well as demographic and occupational characteristics.

## 2. Materials and Methods

### 2.1. Participants

The study was conducted between May and August 2025. Participants were consecutively recruited among PTs working at the Veruno Institute of the Istituti Clinici Scientifici Maugeri (Gattico-Veruno, Novara, Italy). Eligible participants were PTs employed on a full-time basis (five working days per week). To ensure the reliability of weekly fatigue monitoring, evaluations were conducted exclusively during a standard, full workweek; in cases of scheduled leave or absence, the assessment was rescheduled to the participants’ next full week of clinical activity. Exclusion criteria included a history of upper limb trauma or orthopedic surgery within the previous six months, chronic orthopedic or neurological conditions affecting manual dexterity, and ongoing pharmacological treatments known to influence motor control. All participants provided written informed consent before participation. The study was conducted in accordance with the principles of the Declaration of Helsinki.

### 2.2. Procedure

After confirming participants’ eligibility, demographic and occupational information were collected using a standardized questionnaire. Variables included age, sex, hand dominance, department of work, years and months of professional experience as a PT, and weekly working hours. Height and weight were measured using a wall-mounted ruler and a standard analog floor scale.

Subsequently, participants completed two self-report questionnaires assessing habitual sleep quality (Pittsburgh Sleep Quality Index, PSQI) and perceived stress (Perceived Stress Scale, PSS) over the previous month to characterize their baseline status. Only after these questionnaires were administered, participants were evaluated by the raters for manual dexterity, MGS, and subjective fatigue. These assessments were conducted under four temporal conditions: at the beginning and end of the work shift (about at 8.30 a.m. and at 4.30 p.m.), on Monday and Friday. This dual approach allowed for the contextualization of acute performance changes within the participants’ broader psychological and physiological habitual status.

All evaluations were performed during working hours in a quiet room within the Neuromotor Department. To standardize positioning, the same chair was used for all assessments to ensure consistent forearm support during strength testing, and a desk placed in front of the chair served as the testing surface according to the published FDT protocol [23]. The testing protocol, including order and rest intervals, was standardized across participants. Assessments were conducted by two PTs who were members of the project team but did not participate as subjects. Due to the nature of the study and the clinical setting, assessor blinding was not feasible. However, to minimize expectation bias and ensure data objectivity, participants were not informed about the specific research hypotheses, and a standardized, highly structured protocol was strictly followed for each measurement. Additionally, the raters followed a scripted instruction set for all participants to maintain procedural consistency.

Participant data were pseudonymized: individual information was recorded in an Excel database using alphanumeric identification codes only, and the file was stored on a password-protected institutional computer accessible exclusively to the research team.

#### 2.2.1. Sleep Quality and Stress Assessment

Sleep quality over the previous month was assessed using the PSQI. This validated questionnaire yields a global score ranging from 0 to 21, with higher scores indicating poorer sleep quality [26]. The PSQI comprises 19 self-rated items that generate seven component scores: subjective sleep quality, sleep latency, sleep duration, habitual sleep efficiency, sleep disturbances, use of sleep medication, and daytime dysfunction. The instrument has demonstrated good internal consistency, test–retest reliability, and construct validity [26]. Participants were instructed to report their habitual sleep patterns over the previous month, including sleep duration, latency, disturbances, and subjective restfulness.

Perceived stress over the preceding month was evaluated using the PSS [27]. The PSS is a 10-item self-report questionnaire designed to measure the extent to which individuals perceive their lives as unpredictable, uncontrollable, and overloaded. Items are rated on a 5-point Likert scale ranging from 0 (never) to 4 (very often), yielding a total score between 0 and 40, with higher scores indicating greater perceived stress. The PSS captures the subjective experience of stress rather than exposure to objective stressors and has demonstrated robust psychometric properties, including excellent test–retest reliability and strong construct validity across diverse populations [27].

All questionnaires were self-administered under the supervision of the raters, who were available to clarify any potential doubts.

#### 2.2.2. Functional Dexterity Assessment

The FDT was used to assess upper-limb manual dexterity. The FDT is a reliable and valid instrument for evaluating fine motor performance in both clinical and healthy populations, demonstrating good test–retest reliability and sensitivity to changes in manual dexterity [23,28]. The test consists of a square pegboard containing 16 holes and an equal number of cylindrical pegs. During the test, participants were instructed to invert all pegs as quickly as possible using one hand at a time, while the examiner recorded task completion time using a stopwatch. Performance accuracy was continuously monitored. A 5 s penalty was applied each time compensatory movements were observed (e.g., excessive forearm supination or use of the board for external support), whereas a 10 s penalty was assigned if a peg was dropped. The net time (in seconds) reflected manual execution speed, while the total score, obtained by adding penalty time to the net time, represented overall dexterity performance, integrating both speed and movement quality.

The FDT was administered to each participant at four time points: before and after the work shift (morning and evening), both at the beginning and at the end of the workweek (Monday and Friday). In accordance with the original protocol, testing was performed first with the dominant hand [23]. All assessments followed standardized procedures using the validated Italian version of the test [28]. Prior to data collection, participants completed one familiarization trial. Raw data included net completion time and penalty occurrences; however, the corrected total score was used for all statistical analyses, as it reflects a global index of manual proficiency.

#### 2.2.3. Maximal Grip Strength Assessment

MGS was assessed bilaterally using a calibrated Jamar^®^ hydraulic hand dynamometer (Sammons Preston Incorporated, Bolingbrook, IL, USA), set at the second handle position. The device is equipped with a peak-hold needle that retains the maximum value until reset, allowing accurate recording of maximal voluntary contraction. Participants were seated comfortably on a chair with a backrest and fixed arm supports, with the forearms fully supported and both feet flat on the floor. Watches and bracelets were removed before testing. Participants were instructed and shown how to correctly grasp the dynamometer. Measurements were performed with the wrist in a neutral position and the elbow flexed at approximately 90°. The examiner ensured correct positioning of the device and provided light support to the dynamometer to prevent extraneous movement without restricting force production.

Each participant performed three maximal voluntary contractions with each hand, alternating sides, with 1 min rest intervals between trials. Standardized verbal encouragement was provided, instructing participants to squeeze as forcefully as possible until the peak-hold needle stopped rising. The mean value of the three trials for each hand was used for statistical analysis.

Hand dynamometer is a widely validated instrument for both clinical and healthy populations, demonstrating excellent test–retest reliability and sensitivity to changes in muscular performance [29,30].

#### 2.2.4. Subjective Fatigue Assessment

To capture perceived fatigue at each assessment, participants completed an 11-point Numerical Fatigue Rating Scale (NFRS) immediately before FDT and grip strength measurement. Participants were asked to rate their current level of fatigue on a scale ranging from 0 (“no fatigue”) to 10 (“worst imaginable fatigue”). This simple and time-efficient tool is widely used to quantify perceived exertion and fatigue in both clinical and occupational contexts, demonstrating excellent test–retest reliability and high sensitivity to short-term changes in subjective tiredness [31,32].

To specifically assess daily perceived fatigue accumulation, participants completed the Patient-Reported Outcomes Measurement Information System Fatigue Short Form 7b (PROMIS-F) [33]. This instrument evaluates the intensity and impact of fatigue on daily activities, concentration, and motivation, capturing the subjective experience of tiredness rather than objective physical performance. Participants completed the questionnaire at the end of the workday on Monday and Friday, reflecting the average level of fatigue experienced during that specific day. The scale consists of seven items rated on a 5-point Likert scale (from 1 = never to 5 = always), yielding a raw total score ranging from 7 to 35, with higher scores indicating greater perceived fatigue. The PROMIS-F has demonstrated high reliability and robust construct validity across both clinical and healthy adult populations [33,34].

### 2.3. Statistical Analysis

All analyses were performed using STATISTICA 7.1 (TIBCO Software Inc., Tulsa, OK, USA). Data are presented as mean ± standard deviation (SD) or standard error (SE). A significance level of *p* < 0.05 was applied for all statistical tests.

#### 2.3.1. Reliability and Measurement Error Analysis

To evaluate the reproducibility of the FDT, a subsample consisting of the first consecutively enrolled participants performed three consecutive trials per hand (dominant and non-dominant) during the same testing session (Monday morning).

Test–retest reliability was assessed using intraclass correlation coefficients (ICC) calculated with a two-way mixed-effects model for single measurements, assuming consistency and the same raters for all participants (ICC_3,1_). ICC values were interpreted according to commonly accepted thresholds: values < 0.50 indicated poor reliability, 0.50–0.75 moderate reliability, 0.75–0.90 good reliability, and > 0.90 excellent reliability [35].

The required sample size for reliability analysis was estimated a priori based on pilot data indicating an expected ICC of approximately 0.90, with a 95% confidence interval width of approximately 0.20. According to methodological recommendations reported by Sartorio et al. [36], a minimum sample size of 21 participants was required to ensure adequate statistical power for reliability assessment.

Measurement error was quantified by calculating the standard error of measurement (SEM) using the following formula:

SEM = SD baseline × √(1 − ICC_3,1_)

providing an estimate of the within-subject variability due to measurement error.

The minimal detectable change at the 90% confidence level (MDC_90_) was then computed to determine the smallest change that can be interpreted as a true change beyond measurement error:

MDC_90_ = SEM × 1.645 × √2.

These indices allow the identification of meaningful changes in manual dexterity over time, separately for the dominant and non-dominant hand.

#### 2.3.2. Within-Subject Analysis of Manual Dexterity and Maximal Grip Strength

To investigate changes in manual dexterity and MGS across the workweek and between hands, a two-way repeated-measures analysis of variance (ANOVA) was performed. The within-subject factors were TIME (four levels: Monday morning, Monday evening, Friday morning, Friday evening) and LIMB (two levels: dominant and non-dominant hand). This design allowed evaluation of the main effects of TIME and LIMB, as well as their interaction (TIME × LIMB). Post hoc pairwise comparisons were conducted using Tukey’s honestly significant difference (HSD) test to identify significant differences between specific temporal conditions. Effect sizes were calculated as Cohen’s d, defined as the mean of the paired differences divided by the standard deviation of those differences, to quantify within-subject changes. Sphericity was assessed for all repeated-measures analyses using Mauchly’s test. Since the assumption was met for all primary variables (all *p* > 0.05), the reported univariate *p*-values were considered valid, and no epsilon corrections (e.g., Greenhouse–Geisser) were required.

As no previous studies have examined FDT performance across multiple time points within the same workweek, sample size estimation was based on the primary a priori contrast between the beginning and the end of the workday, which was considered clinically meaningful. Based on previous occupational and fatigue-related studies, a conservative mean difference of 3 s and a standard deviation of 5 s were assumed [11]. Sample size was estimated using Cohen’s approach for paired measurements [37], assuming a two-sided α = 0.05, a statistical power of 90%, and a conservative within-subject correlation of less than 0.5. Under these assumptions, the required sample size was set at 42 participants (rounded up).

#### 2.3.3. Correlation Analysis

Spearman’s rank correlation coefficients (ρ) were calculated to examine associations among manual dexterity (FDT), MGS, subjective fatigue (assessed via NFRS and PROMIS-F), sleep quality (PSQI), and perceived stress (PSS), as well as participants’ demographic and occupational characteristics (sex, weekly working hours, and working experience). Spearman’s ρ was selected to account for potential non-normal distributions and to provide a robust measure of monotonic relationships between variables. To reduce the risk of type I error due to multiple comparisons, *p*-values were adjusted using the False Discovery Rate correction (Benjamini–Hochberg procedure).

## 3. Results

A total of 46 full-time PTs was initially screened for eligibility. Three candidates were excluded as they were employed on a part-time basis (morning shift only), which prevented the completion of the end-of-shift assessments. All remaining eligible PTs (*n* = 43: 25 females, 18 males) agreed to participate, resulting in a 100% acceptance rate among eligible staff. 

Descriptive statistics for demographic and occupational characteristics, as well as sleep quality and perceived stress questionnaires, expressed as mean ± SD, are reported in Table 1. Overall, participants showed good sleep quality and moderate levels of perceived stress. Regarding manual dexterity, the FDT performed on Monday morning showed mean values of 21.81 ± 5.24 s for the dominant hand and 24.12 ± 5.03 s for the non-dominant hand.

### 3.1. Reliability and Measurement Error Outcomes

Intra-rater reliability analysis of the FDT, conducted on the first 23 participants included in the study, demonstrated excellent reliability. The ICC_3,1_ was 0.95 (95%CI: 0.91–0.98) for the dominant hand and 0.93 (95%CI: 0.86–0.97) for the non-dominant hand, indicating high measurement consistency. The corresponding SEMs were 0.65 s and 0.94 s, respectively. The MDC_90_ was 1.51 s for the dominant hand and 2.19 s for the non-dominant hand, representing the smallest real change beyond measurement error.

Descriptive statistics for the subsample of participants included in the reliability analysis, expressed as mean ± SD, are also reported in Table 1.

### 3.2. Within-Subject Outcomes of Manual Dexterity and Maximal Grip Strength 

**Figure 1:**
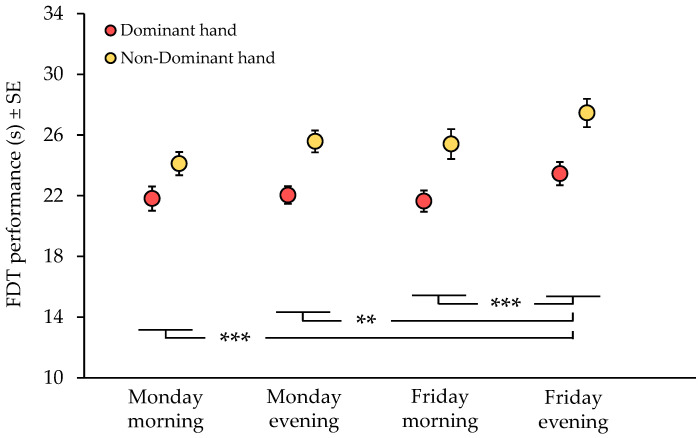
FDT performance across temporal conditions. PTs were tested on Monday and Friday, both at the beginning and at the end of the workday, for the dominant (red circles) and non-dominant (yellow circles) hand. **, *p* < 0.005; ***, *p* < 0.0005.

The two-way repeated-measures ANOVA (Figure 1) revealed a significant main effect of limb dominance on manual dexterity (ANOVA, F(1, 82) = 13.25, *p* < 0.01), with the dominant hand consistently performing faster than the non-dominant hand. A significant main effect of time was also observed (ANOVA, F(3, 246) = 9.77, *p* < 0.01), indicating variations in manual dexterity throughout the workweek. Post hoc comparisons showed that FDT completion times were significantly slower on Friday evening compared with all other time points (*p* < 0.01), suggesting an end-of-week decline in fine motor performance. Specifically, completion time on Friday evening was significantly longer compared with Monday morning (mean difference = 2.54 s, 95%CI: 1.24–3.84, *p* < 0.001, d = 0.50), Monday evening (mean difference = 1.61 s, 95%CI: 0.31–2.90, d = 0.40), and Friday morning (mean difference = 1.89 s, 95%CI: 0.59–3.19, d = 0.43). Importantly, the mean difference between Friday evening and Monday morning exceeded the MDC_90_ thresholds for both the dominant and non-dominant hands (MDC_90_ = 1.51 s and 2.19 s, respectively), while comparisons between Friday evening and Monday evening or Friday morning exceeded the MDC_90_ threshold for the dominant hand only. At the individual level, declines in dexterity exceeding the MDC_90_ from Monday morning to Friday evening were observed in 62.79% (*n* = 27) of participants for the dominant hand and in 67.44% (*n* = 29) for the non-dominant hand. No significant time × limb interaction was found (ANOVA, F(3, 246) = 1.51, *p* = 0.21), indicating that temporal changes in FDT performance were comparable between the dominant and non-dominant hands.

Conversely, for MGS, no significant main effects of limb dominance (ANOVA, F(1, 82) = 0.53, *p* = 0.47), time (ANOVA, F(3, 246) = 0.74, *p* = 0.53), or time × limb interaction (ANOVA, F(3, 246) = 1.24, *p* = 0.30) were found, suggesting stable maximal performance across the workweek and between limbs (detailed numerical values for these non-significant findings are provided in Table 2).

### 3.3. Correlation Analysis Outcomes

**Figure 2:**
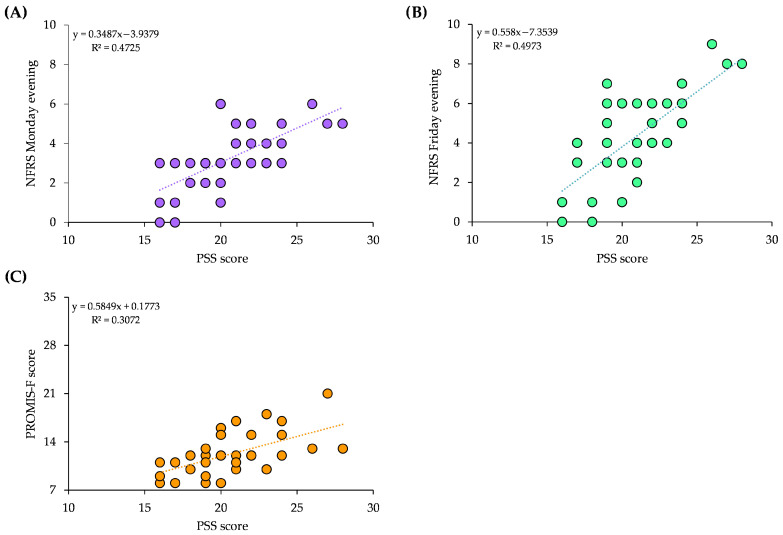
Correlations between stress and fatigue. (**A**) Correlation between perceived fatigue on Monday evening by NFRS and the perceived stress over the preceding month by PSS. (**B**) Correlation between perceived fatigue on Friday evening by NFRS and the perceived stress over the preceding month by PSS. (**C**) Correlation between daily perceived fatigue accumulation by PROMIS-F and the perceived stress over the preceding month by PSS.

Spearman’s correlation analysis demonstrated strong and statistically significant associations between perceived stress (PSS) and subjective fatigue (Figure 2). Perceived stress was strongly correlated with Monday evening fatigue as assessed by the NFRS (ρ = 0.731, *p* < 0.001) and the PROMIS-F (ρ = 0.598, *p* < 0.001). Similarly, a strong association was observed between perceived stress and Friday evening fatigue measured using the NFRS (ρ = 0.612, *p* < 0.001). Interestingly, sleep quality (PSQI) did not correlate with any other outcome measure after FDR correction. Overall, these findings indicate that higher perceived stress, but not poorer sleep quality, was consistently associated with greater fatigue levels, suggesting a meaningful link between psychosocial strain and early-week fatigue perception among physiotherapists.

Sex showed a strong and consistent correlation with grip strength across all time points (at least ρ = 0.798, *p* < 0.001), confirming markedly higher strength values in males compared with females. Conversely, no significant associations were observed between sex and FDT performance, either at individual time points or in weekly changes, suggesting that sex did not substantially influence manual performance dynamics over the week.

In addition, neither working experience, weekly working hours, nor weekly changes in manual dexterity were significantly associated with any of the measured variables.

## 4. Discussion

This study investigated manual performance in PTs across different times of the day and the working week, providing novel insight into how fine motor function and strength evolve under routine occupational demands. The main finding was a progressive and clinically meaningful worsening of functional manual dexterity toward the end of the workweek, as assessed by the FDT, whereas MGS remained stable across all time points. This dissociation might suggest that work-related fatigue in PTs selectively affects coordination and precision-dependent motor functions rather than peak force-generating capacity, at least at a functional level.

The observed increase in FDT completion times on Friday evening exceeded the MDC_90_ threshold for the dominant hand, supporting the interpretation of a true decline in fine motor performance rather than random measurement variability. While the MDC_90_ is primarily an individual-level metric, its use here serves as a conservative contextual benchmark to support the interpretation that the observed mean decline reflects a meaningful trend rather than random measurement variability. Importantly, this deterioration was evident in both hands and was not confined to comparisons within the same day. Manual dexterity on Friday evening was worse not only relative to Friday morning but also compared with Monday morning and evening, indicating that the observed changes reflect cumulative rather than transient effects.

The excellent intra-rater reliability and low measurement error observed for the FDT further reinforce its suitability for detecting subtle, work-related changes in fine motor performance. Intra-rater consistency was excellent for both the dominant and non-dominant hand, with low SEM and MDC_90_ values. These reliability indices closely align with those previously reported in healthy adult populations, supporting the robustness and reproducibility of the FDT even when applied in professional cohorts exposed to cumulative occupational load [23,36]. The use of the total penalized score, rather than raw completion time, was preferred as it integrates both speed and movement quality. In clinical tasks, neuromuscular fatigue often manifests as a trade-off between speed and accuracy; therefore, incorporating penalties for dropped pegs or compensatory movements may provide a more comprehensive reflection of functional manual proficiency. Moreover, FDT scores at baseline were consistent with normative data for healthy adults of similar age, indicating that participants exhibited typical levels of manual dexterity and strengthening the interpretation that the end-of-week decline reflects fatigue-related effects rather than pre-existing motor impairment [23,36].

As expected, the dominant hand consistently outperformed the non-dominant hand, in line with well-documented asymmetries in motor control and movement efficiency [38,39]. This performance pattern is consistent with established neuromotor principles, suggesting that the cohort’s motor behavior reflects known differences in prehensile synergies and control strategies between the dominant and non-dominant hand [39,40].

More importantly, manual dexterity was not stable across the week. Performance remained relatively preserved early in the week, with no intra-day differences observed, but progressively declined toward its end. The magnitude of the difference observed between Friday morning and Friday evening was comparable to reductions reported in other healthcare professions, such as dental hygienists, where significant late-week reductions in dexterity performance were observed [11]. While those studies primarily focused on intra-day changes, the present findings extend existing evidence by demonstrating that late-week manual dexterity is impaired not only within the same day but also relative to performance at the beginning of the week.

The observed temporal pattern suggests that, while overnight recovery appears sufficient at the beginning of the week, it proves to be inadequate to counterbalance the cumulative weekly load of physically and cognitively demanding tasks, highlighting a clear cumulative fatigue effect by the end of the week. Activities requiring high precision, rapid adjustments, and coordinated finger movements are particularly vulnerable to this cumulative fatigue [1,19]. While the physical demands of occupational therapy and high-performance sports differ significantly, these elite contexts provide illustrative models for the general principles of fine motor fatigue. For instance, in elite soccer, constant technical adjustments under pressure drive neuromuscular and mental exhaustion [41], while in table tennis, cumulative mental exertion significantly degrades distal accuracy and speed despite stable physiological output [42]. Such parallels reinforce the idea that fine motor control is particularly prone to systemic occupational fatigue.

The absence of interaction between time and limb indicates that both hands deteriorated similarly over time, despite the non-dominant hand showing a larger absolute slowdown. This pattern is compatible with non-limb-specific mechanisms, potentially pointing toward factors such as central fatigue, reduced attentional resources, or increased sensorimotor noise rather than localized overuse or unilateral overload [1,13]. Such interpretations would be consistent with models of fatigue that emphasize central nervous system involvement and diminished motor precision under prolonged cognitive and physical demands [5,13,43].

In contrast, MGS showed no significant variation over time or between limbs. This stability likely indicates that the mechanisms typically responsible for acute reductions in maximal voluntary force, such as substantial inhibition of central motor drive or reduced recruitment of high-threshold motor units, were not sufficiently engaged by the occupational demands experienced by the participants. This interpretation is consistent with previous evidence indicating that maximal force production is relatively insensitive to moderate levels of occupational and central fatigue, particularly when workloads do not involve sustained maximal contractions [1]. As previously noted in a study on healthcare workers, typical occupational physical demands may be insufficient to induce measurable peripheral fatigue in terms of peak force, even though mental and physical fatigue can significantly affect tasks requiring endurance, motor control, or complex cognitive processing [44].

Importantly, manual dexterity tasks are highly dependent on force steadiness and fine motor control rather than on maximal force-generating capacity [45]. Previous work has shown that performance in manual dexterity tests is strongly predicted by force steadiness during low-force isometric contractions, particularly in middle-aged adults [46,47,48]. Accordingly, coordination-dependent tasks may deteriorate even when maximal force output is preserved, reflecting distinct underlying fatigue mechanisms. In fact, maximal force production depends on different physiological mechanisms and is often preserved until fatigue reaches a magnitude sufficient to impair voluntary activation or peripheral contractile function [49]. The divergence between MGS and FDT performance reinforces the notion that fine motor tests are more sensitive to early functional changes associated with occupational fatigue and reduced motor control precision [9,19,25]. While MGS remains a valuable measure of general muscular capacity [50,51], it may not capture subtle declines in functional readiness that are relevant to clinical performance. In contrast, dexterity-based measures appear to integrate central, sensorimotor, and attentional processes, potentially making them more sensitive to fatigue-related changes in work environments with sustained cognitive-motor demands [19,49].

The role of individual factors was also explored to clarify the origin of the observed fatigue. In this cohort, only a minority of participants reported poor sleep quality, and no significant association emerged between PSQI scores and either manual performance or subjective fatigue. This lack of association suggests that the perceived exhaustion and the decline in dexterity are not primarily driven by sleep-related factors. Instead, the fatigue profile observed in this sample may reflect a typical occupational pattern. While sleep quality was generally adequate, the load of the PTs’ work may prevent overnight recovery from being sufficient to fully reset the system by the end of the workweek. This interpretation aligns with the finding that performance impairments typically emerge from the accumulation of daily workloads rather than isolated sleep disturbances, unless marked sleep deprivation is present [52,53].

Contrary to sleep quality, we observed significant associations between perceived stress and subjective fatigue accumulated over the workday, already apparent earlier in the workweek. This suggests that perceived stress may act as an immediate driver of fatigue, predominantly reflecting centrally mediated or psychological components. This pattern could relate to the anticipation of high weekly job demands or to the carry-over of professional stress that a weekend of rest may not fully resolve. PTs routinely manage patients with complex and chronic conditions over prolonged periods, exposing them to sustained physical and mental demands that generate considerable work-related stress, independent of absolute physical workload [54]. High mental demands, time pressure, and substantial clinical responsibilities expose physiotherapists to chronic psychosocial stress, which has been linked to emotional exhaustion, reduced professional effectiveness, and a higher prevalence of musculoskeletal pain and sleep disturbances among healthcare professionals [54,55,56]. Given that low job satisfaction and mental health problems can reduce professional effectiveness in PTs [56], concurrent monitoring of perceived stress alongside objective dexterity measures such as the FDT may provide a more comprehensive understanding of functional readiness and occupational well-being.

Furthermore, no significant associations were observed between age and FDT performance, likely reflecting the relatively narrow age range of the sample. This aligns with prior research showing that marked declines in manual dexterity typically emerge only after age 60 [57,58]. Similarly, no sex-related differences were detected, consistent with earlier findings showing comparable dexterity performance between males and females across adult age groups [58]. Interestingly, previous work has suggested that anthropometric factors influence dexterity performance more strongly than sex per se, particularly in the non-dominant hand, highlighting the importance of incorporating simple anthropometric measures into hand function assessments in occupational settings [59].

Several limitations should be acknowledged. First, the sample size was modest, and we focused on short-term fluctuations within a single workweek, without addressing potential long-term fatigue accumulation.

Regarding the methodology, this study was not designed to validate the FDT as a direct measure of NMF; thus, conclusions should be interpreted with caution. Since the FDT provides only an indirect assessment, integrating objective measures such as EMG, grip force variability, or kinematic analyses would be necessary to clarify underlying mechanisms. Furthermore, as there is no consensus on error-based time penalties, the choice to rely on penalized FDT scoring instead of raw completion time raises the possibility that the specific scoring method employed may have influenced the observed outcomes and their sensitivity to subtle fatigue-related changes [36,60]. Additionally, as neither assessors nor participants were blinded, the potential for expectation and observer bias cannot be entirely excluded, although standardized protocols and scripted instructions were employed to mitigate these effects.

Concerning psychophysiological factors, sleep quality and perceived stress were assessed via subjective self-reports reflecting habitual status over the previous month. While these instruments do not capture acute daily variations, they allowed for the characterization of the participants’ baseline allostatic load, which influences neuromuscular resilience and recovery [61]. However, the lack of concurrent objective measures (e.g., actigraphy or sleep diaries) limits the ability to establish direct causal links between nightly sleep and daily performance. Similarly, while sex was strongly correlated with absolute strength, our analysis did not stratify fatigue dynamics by sex due to the limited sample size. Given the known physiological differences between males and females, the lack of sex-specific stratification represents a limitation that future studies with larger cohorts should address.

Finally, daily workload intensity and task composition were not formally quantified. In particular, the study did not account for non-clinical hand use, such as smartphone and computer interaction after work. Given the high prevalence of digital device usage, which imposes additional submaximal loads on the forearm musculature, the cumulative effect of these activities could represent a confounding variable. Future studies should incorporate logs or objective monitoring of extracurricular hand use to better isolate the specific impact of clinical activity on manual performance.

## 5. Conclusions

This study demonstrates that PTs experience a progressive decline in manual dexterity over the course of a typical workweek, while MGS remains stable. The observed deterioration highlights that cumulative occupational demands appear to selectively impair coordination- and precision-dependent motor functions rather than peak force production. These findings underscore the sensitivity of fine motor assessments, such as the FDT, in capturing early functional decrements compatible with central and sensorimotor fatigue, even in the absence of overt muscular weakness or poor sleep quality. Perceived stress emerged as an important contributor to subjective fatigue, suggesting that psychosocial strain influences how fatigue is experienced.

From a practical perspective, routine monitoring of manual dexterity alongside stress assessment could provide a more comprehensive picture of PTs’ functional readiness and occupational well-being. Such an approach could inform workload management, task scheduling, and recovery strategies, supporting effective clinical practice. Future research should investigate whether similar patterns emerge under heavier workloads, in different healthcare professions, or over longer observation periods, and should explore the relationship between dexterity changes, task demands, stress, and clinical outcomes. Integrating FDT performance with objective physiological measures, recovery metrics, and psychosocial assessments may clarify whether observed changes reflect specific neuromuscular fatigue, central fatigue, or broader sensorimotor and psychological adaptations. Longitudinal studies extending beyond a single workweek, ideally in larger and more heterogeneous cohorts, are warranted to provide insight into cumulative fatigue and inform preventive strategies such as task rotation, workload redistribution, and optimized recovery periods, ultimately contributing to both clinician performance and patient safety.

## Figures and Tables

**Table 1:** Characteristics of the total sample of physiotherapists (*n* = 43) and of the subgroup in reliability assessment (*n* = 23).

Characteristics of PTs	Total Sample (*n* = 43)	Reliability Subgroup (*n* = 23)
Demographical and occupational		
Age (years)	37.72 ± 11.94	42.52 ± 11.70
Sex (Female/Male)	25/18	12/11
Height (cm)	170.80 ± 8.29	174.06 ± 9.96
Weight (kg)	66.00 ± 12.45	69.31 ± 13.95
Limb dominance (Right/Left)	(42/1)	(23/0)
Working experience (years)	7.93 ± 9.13	9.90 ± 9.75
Weekly working hours	36.95 ± 3.83	37.00 ± 4.06
Subjective questionnaires		
Pittsburgh Quality Index	3.85 ± 1.84	3.50 ± 2.25
Perceived Stress Scale	20.75 ± 2.67	20.31 ± 2.89

**Table 2:** Mean values (±SD) for Maximal Grip Strength in the total sample stratified by hand.

	All Sample (*n* = 43)	
	Dominand Hand	Non-Dominant Hand
MGS Monday Morning (kg)	44.72 ± 11.89	41.35 ± 9.79
MGS Monday Evening (kg)	44.16 ± 12.32	41.73 ± 13.26
MGS Friday Morning (kg)	45.44 ± 13.26	42.44 ± 11.63
MGS Friday Evening (kg)	44.04 ± 12.85	42.12 ± 10.88

## Data Availability

The datasets used during the current study are available from the corresponding author on reasonable request due to privacy reasons.

## Abbreviations

The following abbreviations are used in this manuscript:

EMG	Electromyographic
FDT	Functional dexterity test
ICC	Intraclass Correlation Coefficients
MDC	Minimal detectable change
MGS	Maximal grip strength
NMF	Neuromuscular fatigue
NFRS	Numerical Fatigue Rating Scale
PROMIS-F	Patient-Reported Outcomes Measurement Information System-Fatigue
PSQI	Pittsburgh Sleep Quality Index
PSS	Perceived stress scale
PTs	Physiotherapists
SEM	Standard error of measurement
